# MOF as an evolutionarily conserved histone crotonyltransferase and transcriptional activation by histone acetyltransferase-deficient and crotonyltransferase-competent CBP/p300

**DOI:** 10.1038/celldisc.2017.16

**Published:** 2017-05-23

**Authors:** Xiaoguang Liu, Wei Wei, Yuting Liu, Xueli Yang, Jian Wu, Yang Zhang, Qiao Zhang, Tieliu Shi, James X Du, Yingming Zhao, Ming Lei, Jin-Qiu Zhou, Jiwen Li, Jiemin Wong

**Affiliations:** 1 Shanghai Key Laboratory of Regulatory Biology, The Institute of Biomedical Sciences and School of Life Sciences, East China Normal University, Shanghai, China; 2 State Key Laboratory of Molecular Biology, CAS Center for Excellence in Molecular Cell Science, Shanghai Institute of Biochemistry and Cell Biology, Chinese Academy of Sciences, University of Chinese Academy of Sciences, Shanghai, China; 3 National Center for Protein Science Shanghai, State Key Laboratory of Molecular Biology, Institute of Biochemistry and Cell Biology, Shanghai Institutes for Biological Sciences, Chinese Academy of Sciences, Shanghai, China; 4 Shanghai Science Research Center, Chinese Academy of Sciences, Shanghai, China; 5 Ben May Department of Cancer Research, The University of Chicago, Chicago, IL, USA; 6 Joint Research Center for Translational Medicine, East China Normal University and Shanghai Fengxian District Central Hospital, Shanghai, China

**Keywords:** crotonylation, acetylation, acylation, CBP/p300, MOF, Esa1, transcription, chromatin

## Abstract

Recent studies indicate that histones are subjected to various types of acylation including acetylation, propionylation and crotonylation. CBP and p300 have been shown to catalyze multiple types of acylation but are not conserved in evolution, raising the question as to the existence of other enzymes for histone acylation and the functional relationship between well-characterized acetylation and other types of acylation. In this study, we focus on enzymes catalyzing histone crotonylation and demonstrate that among the known histone acetyltransferases, MOF, in addition to CBP and p300, also possesses histone crotonyltransferase (HCT) activity and this activity is conserved in evolution. We provide evidence that CBP and p300 are the major HCTs in mammalian cells. Furthermore, we have generated novel CBP/p300 mutants with deficient histone acetyltransferase but competent HCT activity. These CBP/p300 mutants can substitute the endogenous CBP/p300 to enhance transcriptional activation in the cell, which correlates with enhanced promoter crotonylation and recruitment of DPF2, a selective reader for crotonylated histones. Taken together, we have identified MOF as an evolutionarily conserved HCT and provide first cellular evidence that CBP/p300 can facilitate transcriptional activation through histone acylation other than acetylation, thus supporting an emerging role for the non-acetylation type of histone acylation in transcription and possibly other chromatin-based processes.

## Introduction

Histone acetylation in lysine residues is an archetype of histone post-translational modification (PTM) that has been extensively studied and shown to have critical roles in the regulation of chromatin structure and function [1, 2]. Histone acetylation is best known for its positive role in transcriptional regulation. It is a reversible PTM for which the molecular system responsible for its establishment, removal and recognition has been well characterized [3–8]. The families of histone acetyltransferases (HATs) are able to catalyze acetylation of histones as well as non-histone substrates, whereas the families of histone deacetylases catalyze the reverse reaction. Histone acetylation neutralizes the positive charge in the lysine residue and is believed to regulate transcription in part through its effect on chromatin structure. In addition, histone acetylation is believed to regulate transcription by providing binding sites for specific reader proteins that bind acetylated histones via structural domains such as bromodomain [9], YEATS [10, 11] or double PHD finger (DPF) [12, 13].

A significant progress in histone modifications in recent years is the finding that in addition to acetylation, lysine residues in histones are also subjected to other types of acylation including malonylation, propionylation, butyrylation, crotonylation and succinylation [14–19]. The identification of these new PTMs strongly implicates a close coupling of cellular metabolism and epigenetic regulation, as their corresponding donors are metabolic intermediates. Although the function of these PTMs remains largely unexplored and the enzymes responsible for their addition and removal remain relatively undefined, limited studies have demonstrated that CBP and p300, two highly related HATs that function as general transcriptional coactivators, are capable of catalyzing propionylation [20, 21], butyrylation [21] and crotonylation [22]. However, biochemical and structural study revealed a reduced p300 enzymatic activity toward acyl-CoA with increased acyl-chain length [23]. GCN5 and PCAF have also been shown to catalyze histone propionylation and butyrylation, but with reduced activities compared with histone acetylation [24, 25]. So far, there is no report for histone acylation by a non-HAT enzyme. The fact that various HATs catalyze both histone acetylation and other types of acylation not only makes the functional studies of these new types of histone PTM difficult but also raises the question on the function of histone acetylation, because much of the *in vivo* functional evidence for histone acetylation comes from gain and loss of functional studies on HATs. In this aspect, histone crotonylation is particularly interesting because it occurs broadly in all core histones [15]. The initial pioneering study has identified 28 lysine crotonylation (Kcr) sites in core histones and demonstrated that histone crotonylation marks either active promoters or potential enhancers in both human somatic and mouse male germ cells [15]. A recent study provides evidence that CBP and p300 can catalyze histone crotonylation and that histone crotonylation is more active than histone acetylation in promoting transcription in *in vitro* transcriptional assay [22]. Notably, the recent findings that the previously identified acetylation readers such as AF9, YEATS2 and Taf14 and double PHD finger proteins MOZ and DPF2 actually exhibit higher affinity for crotonylation than acetylation and other types of acylation have provided compelling evidence for a distinct role of histone crotonylation in transcription [26–29]. However, as the cellular concentration of crotonyl-CoA, the donor for crotonylation, is estimated to be about three orders of magnitude lower than acetyl-CoA [22], the physiological relevance of this modification in transcription remains to be demonstrated. Furthermore, as histone crotonylation is conserved in evolution [15], additional histone crotonyltransferase(s) (HCT) must exist, because yeast lacking a CBP/p300 homolog yet is positive for histone crotonylation.

Here we report that, in addition to CBP/p300, MOF also catalyzes histone crotonylation. Furthermore, Esa1, the yeast homolog of mammalian MOF, is responsible for bulk histone crotonylation in yeast, thus identifying MOF as an evolutionarily conserved HCT. We also demonstrate that CBP and p300 are the major HCTs in mammalian cells. Importantly, we have generated a novel p300 and the equivalent CBP mutant with deficient HAT but competent HCT activity. Using these mutants, we demonstrate that CBP/p300 can enhance transcriptional activation without catalyzing histone acetylation and that the transcriptional activation by the mutant CBP/p300 correlates with enhanced promoter crotonylation and recruitment of histone crotonylation reader proteins.

## Results

### MOF also possesses HCT activity

Although CBP and p300 have been shown to catalyze histone crotonylation, additional HCT(s) must exist as histone crotonylation was detected in yeast, which lacks a CBP/p300 homolog [15]. Given the similarity of crotonyl-CoA to acetyl-CoA and the observed HCT activity for CBP/p300, we hypothesized that additional HAT protein(s) may possess HCT activity. To test this idea, we first characterized the commercially available antibodies against pan-crotonylated lysine (Kcr) [50]. As shown in Supplementary Figure S1A, a monoclonal and a polyclonal pan-Kcr antibody recognized specifically the synthetic crotonylated but not acetylated H3 peptide. Furthermore, when applied for immunofluorescent (IF) staining analysis, both antibodies strongly stained HeLa cells treated with sodium crotonate (NaCr) and weakly the control HeLa cells (Supplementary Figure S1B). Note that the increased Kcr signal was detected primarily in the nucleus, suggesting that crotonylation mainly occurs on nuclear proteins. As a further test for the specificity of the antibodies, we prepared core histones from HeLa cells treated with various concentrations of NaCr and analyzed the effect on histone modifications by western blotting (WB) analysis. We found that NaCr treatment led to a dose-dependent increase of histone crotonylation in H3, H2A/H2B and H4 (Supplementary Figure S1C). However, NaCr treatment only had a very mild effect on histone acetylation and butyrylation and no effect on methylation at various sites, especially when NaCr was below 10 mm. These results demonstrate that the pan-Kcr antibody is highly specific and suitable for IF staining analysis of Kcr in cells.

**Figure 1:**
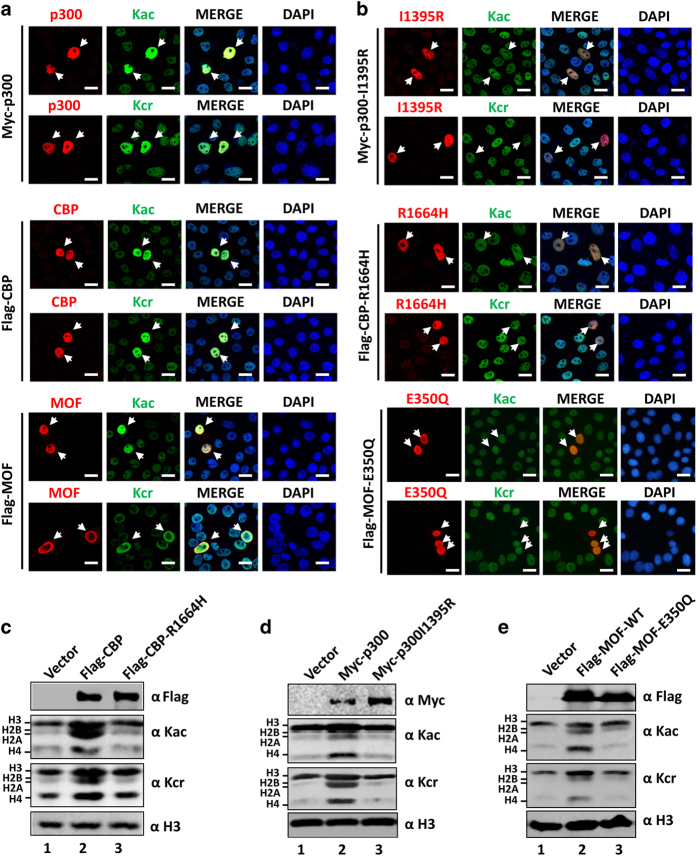
CBP, p300 and MOF possess histone crotonyltransferase (HCT) activities. (**a**) Examining the HCT activity for ectopically expressed CBP, p300 and MOF by immunofluorescent (IF) staining assay using pan-Kcr antibody. HeLa cells were transfected with expression plasmids for CBP, p300 or MOF and the histone acetyltransferase (HAT) and HCT activities were detected by IF staining using pan-Kcr or pan-Kac antibody as indicated. Scale bar, 20 μm. (**b**) IF staining assay showing that the CBP, p300 and MOF mutants inactive for HAT activity were also inactive in catalyzing crotonylation. Scale bar, 20 μm. (**c**–**e**) Western blotting (WB) analysis of core histones derived from HeLa cells transfected with or without the wild-type or mutant CBP (**c**), the wild-type or mutant p300 (**d**) or the wild-type or mutant MOF (**e**).

We next tested within nuclear HAT proteins for potential HCT enzymes by transient transfection of each HAT in HeLa cells at a low efficiency followed by IF staining using anti-pan Kcr antibody. As a positive control, we performed IF staining using anti-pan Kac antibody. As shown in Figure 1a and Supplementary Figure S2, IF staining using anti-pan Kac antibody revealed elevated levels of acetylation in HeLa cells transfected with each of the nuclear HATs, in agreement with their well-known HAT activity. Consistent with the previous report [22], IF staining using anti-pan Kcr antibody demonstrated that CBP- and p300-transfected cells exhibited substantially increased levels of Kcr (Figure 1a), whereas no increase of Kcr was observed in cells transfected with GCN5, PCAF, TIP60 and HBO1 (Supplementary Figure S2). To our delight, we observed that MOF-transfected cells also exhibited substantially increased levels of Kcr (Figure 1a). Thus, in addition to CBP and p300, MOF also possesses HCT activity.

### The same catalytic center for HAT and HCT activities

We next determined whether the same catalytic center is responsible for both HCT and HAT activity of CBP, p300 and MOF. We tested HAT-deficient CBP and p300 mutants [30] and HAT-deficient MOF mutant [31] for their ability to catalyze crotonylation by IF staining. As shown in Figure 1b, these mutants were defective not only in acetylation but also crotonylation, indicating that the same catalytic center is involved in histone acetylation and crotonylation. To test this further, we used WB analysis to compare the ability for both wild-type and mutant proteins to catalyze histone crotonylation under the conditions that more than 90% cells were transfected (data not shown). The results in Figure 1c and d showed that transfection of wild-type CBP and p300 resulted in increased levels of both histone acetylation and crotonylation, whereas transfection of the CBP-R1664H and p300 I1395R mutants failed to do so. Similarly, we found that transfection of wild-type but not the MOF-E350Q mutant substantially elevated the levels of histone crotonylation, especially crotonylation of H2A/H2B and H4 (Figure 1e). Together, we conclude that the same enzymatic reaction center is used for CBP, p300 and MOF to catalyze histone acetylation and crotonylation.

### MOF can catalyze histone crotonylation at multiple sites

**Figure 2:**
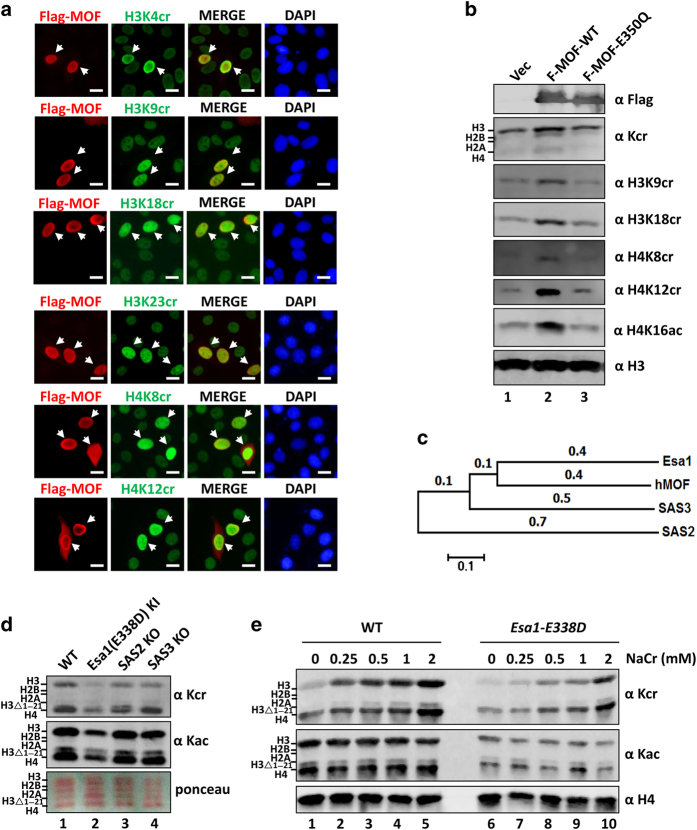
Characterization of the histone crotonyltransferase (HCT) activity of MOF and its yeast homolog Esa1. (**a**) Immunofluorescent (IF) assay showing that MOF can catalyze histone crotonylation at multiple sites. HeLa cells were transfected with Flag-MOF and IF assays were performed with site-specific crotonylated histone antibodies as indicated. (**b**) Western blotting (WB) analysis showing that MOF can catalyze histone crotonylation at multiple sites. (**c**) The phylogenetic tree of mammalian MOF and its related yeast histone acetyltransferase (HAT) proteins. (**d**) WB analysis of core histones derived from the wild-type, *Esa1*(E338D), *sas2*∆ and *sas3*∆ yeast cells. Note the substantially reduced levels of crotonylation and acetylation in core histones derived from *Esa1*(E338D) mutant but not *sas2*∆ and *sas3*∆ cells. Also shown were core histones revealed by ponceau S staining. (**e**) Sodium crotonate treatment resulted in substantially increased levels of histone crotonylation in the wild-type but not *Esa1*(E338D) mutant cells. Yeast cells were treated with an increasing concentration of NaCr for 4 h and core histones were prepared and subjected to WB analysis using pan-crotonylated lysine, pan-acetylated lysine or H4 antibody as indicated.

To characterize in more detail histone crotonylation by MOF, we made use of multiple site-specific histone crotonylation antibodies and IF staining assay. As shown in Figure 2a, MOF was able to catalyze crotonylation on histone H3 at lysine residues 4, 9, 18 and 23 and histone H4 at lysine 8 and 12. By WB analysis, we also verified the ability for wild-type but not the E350Q mutant to catalyze histone crotonylation at multiple sites (Figure 2b). As a positive control, we confirmed MOF catalyzes H4K16 acetylation. Similarly, we found that CBP and p300 were able to catalyze crotonylation at these sites as well (Supplementary Figure S3). Thus, our analysis with commercial antibodies revealed that, like CBP and p300, MOF can catalyze histone crotonylation at multiple sites.

### The MOF homolog Esa1 is a yeast HCT

Histone crotonylation was reported in yeast [15]. Our finding that MOF is an HCT raised the possibility that its yeast homolog might be responsible for histone crotonylation in yeast. Phylogenetic analysis indicates that the closest yeast homolog of human MOF is Esa1, the only essential HAT in budding yeast [32, 33] (Figure 2c). To test whether Esa1 is an HCT in yeast, we prepared core histones from yeast strains of wild-type, *sas2*∆, *sas3*∆ and *eas1*-E338D mutants. Unlike the other two HATs, *Esa1* is an essential gene in yeast. The E338D mutant is defective in HAT activity but is sufficient to support viability of yeast cells [33]. As shown in Figure 2d, a marked reduction of histone crotonylation was observed in *esa1*(E338D) mutant but not in *sas2*∆ or *sas3*∆ strains. As expected, reduced levels of histone acetylation were also observed in *esa1*(E338D) mutant. In further support of Esa1 as an HCT in yeast, we found that while addition of sodium crotonate to culture medium substantially enhanced histone crotonylation in wild-type yeast in a dose-dependent manner, the effect was much reduced in *esa1*(E338D) mutant cells (Figure 2e). Together, these data support histone crotonylation in yeast as an enzymatic reaction and Esa1 as the HCT responsible for bulk histone crotonylation in yeast.

### CBP and p300 are likely the major HCTs in mammalian cells

**Figure 3:**
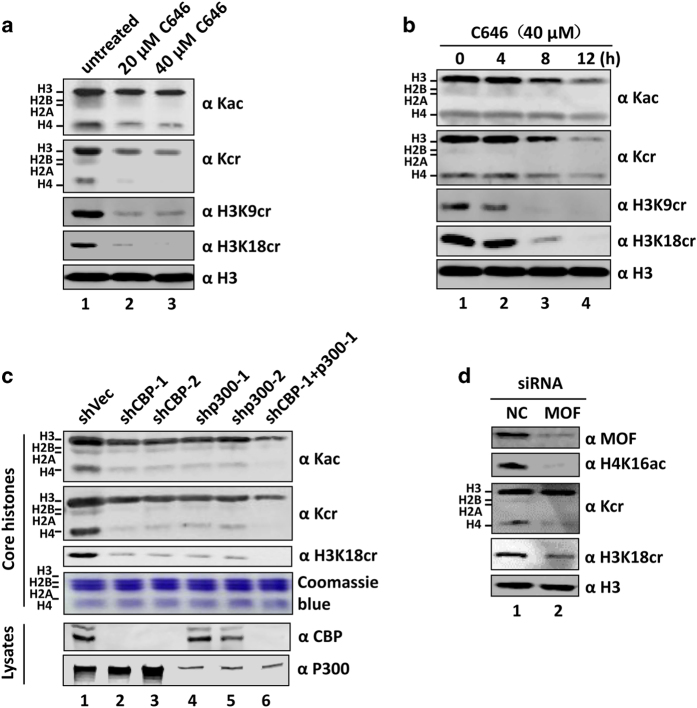
p300 and CBP are major histone crotonyltransferase (HCT) activities in mammalian cells. (**a**) HeLa cells were treated with different concentrations of CBP/p300 selective inhibitor C646 overnight and core histones were prepared and subjected to western blotting (WB) analysis. (**b**) HeLa cells were treated with 40 μm C646 for 0, 4, 8 and 12 h and core histones were prepared and subjected to WB analysis. (**c**) 293T cells were treated with different short hairpin RNAs (shRNAs) against CBP or p300 alone or both for 2 days and core histones were prepared and subjected to WB analysis as indicated. The knockdown efficiency of CBP and p300 was confirmed by WB analysis of cell lysates. (**d**) HeLa cells were treated with small interfering RNA (siRNA) against MOF for 2 days and core histones were prepared and subjected to WB analysis as indicated. The knockdown efficiency of MOF was confirmed by WB analysis of cell lysates.

Having established that CBP, p300 and MOF have intrinsic HCT activity, we next assessed whether they are responsible for bulk histone crotonylation in mammalian cells. To this end, we first made use of a selective CBP/p300 inhibitor C646 [34]. We found this compound inhibited both histone acetylation and crotonylation in HeLa cells in a dose- and time-dependent manner (Figure 3a and b). At a concentration of 40 μM, C646 compound was able to substantially inhibit histone crotonylation in all cell lines tested (Figure 3a and b, and Supplementary Figure S4), suggesting that CBP/p300 is likely the HCT enzymes responsible for bulk histone crotonylation in mammalian cells. In support of this idea, we found that knockdown of either CBP or p300 alone by short hairpin RNA (shRNA) in 293T cells led to substantially reduced levels of histone crotonylation (Figure 3c). Furthermore, simultaneous knockdown of both CBP and p300 resulted in almost a complete loss of H3K18cr and H4 and H2A/H2B crotonylation (Figure 3c). On the other hand, knockdown of MOF by small interfering RNA (siRNA) resulted in a moderate reduction of crotonylated H3 and H4 (Figure 3d). However, a substantial reduction of H4K16 acetylation was observed on MOF knockdown, indicating the observed moderate reduction of crotonylated H3 and H4 was unlikely a result of inefficient MOF knockdown. Taken together, we conclude that CBP and p300 are likely the major HCT enzymes responsible for bulk histone crotonylation in mammalian cells.

### Generation of p300 I1395G and CBP I1432G mutants with HCT but impaired HAT activity

**Figure 4:**
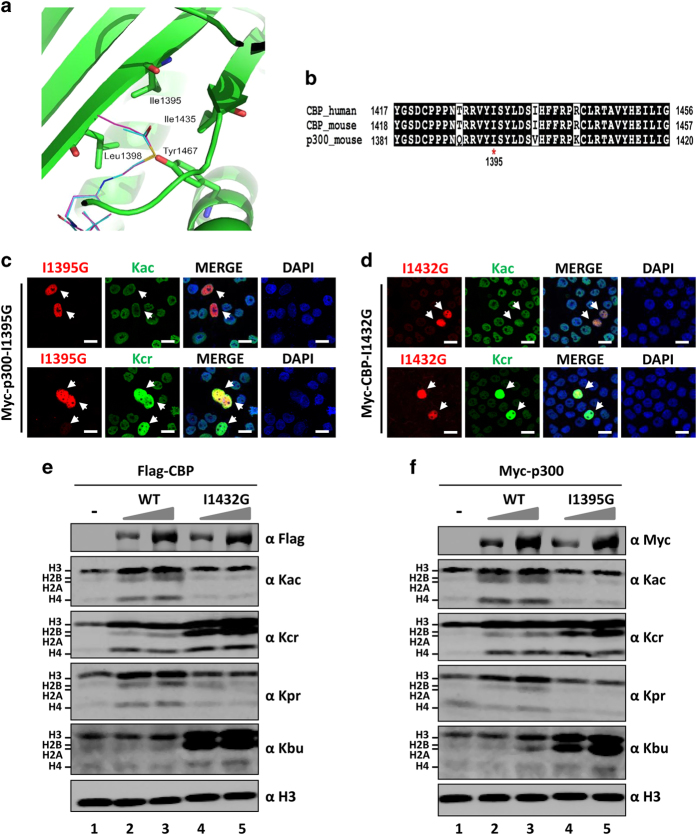
Identification of novel CBP and p300 mutants with deficient histone acetyltransferase (HAT) and competent histone crotonyltransferase (HCT) activity. (**a**) Diagram illustrating the acetyl-coA binding pocket of p300. PDB 3BIY. (**b**) The acetyl-coA binding pocket sequence alignment between CBP and p300. (**c**) Immunofluorescent (IF) staining assay showing that the p300 I1395G mutant was inactive for acetylation but active for crotonylation. Scale bar, 20 μm. (**d**) IF staining assay showing that the CBP I1432G mutant was inactive for acetylation but active for crotonylation. Scale bar, 20 μm. (**e**) Western blotting (WB) analysis of core histones derived from WT or I1432G mutant CBP expressed 293T cells. Lanes 2 and 4, 2 μg plasmids; lane 3 and 5, 4 μg plasmids. (**f**) WB analysis of core histones derived from WT or I1395G mutant p300 expressed 293T cells. Lanes 2 and 4, 2 μg plasmids; lane 3 and 5, 4 μg plasmids.

The fact that MOF, CBP and p300 catalyze both histone acetylation and crotonylation raises a fundamental question as to what extent the function of these HATs in transcription is dependent on their HAT and/or HCT activity. It also makes the functional characterization of histone crotonylation technically challenging. To study the role of HCT activity in transcription, we thought it is necessary to generate an MOF or CBP/p300 mutant with active HCT but inactive HAT activity. As we failed to detect HCT activity for recombinant MOF *in vitro* (data not shown), we focused our effort on CBP and p300 because the p300 HAT domain alone is able to catalyze histone crotonylation *in vivo* and *in vitro* (Supplementary Figure S5). Given that crotonyl-CoA is bulkier than acetyl-CoA, we hypothesized that changing the size of the pocket for binding acetyl-CoA might alter the donor selectivity and therefore differentially affect their HCT or HAT activity. On the basis of the reported p300 and acetyl-CoA co-structure [35], we focused on mutation of isoleucine residue 1395, whose position in the structure is likely to determine the length of the acyl moiety suitable for the pocket (Figure 4a). Sequence alignment of CBP and p300 revealed that this amino acid and the entire region are highly conserved in evolution and between CBP and p300 (Figure 4b). Not surprisingly, we found that substitution of I1395 with other amino acids either maintains both activities or results in a partial or complete loss of both HAT and HCT activities, including acetylation and crotonylation of non-histone proteins (data not shown, also see Supplementary Figure S6A). Notably, we obtained one p300 mutant (I1395G) that is defective in HAT but maintains the HCT activity as shown by IF staining analysis in Figure 4c (also see Supplementary Figure S6B for acetylation and crotonylation of non-histone proteins). Furthermore, the equivalent I1432G mutation in CBP (Figure 4b) also resulted in a CBP mutant with impaired HAT but active HCT activity (Figure 4d). The feature of these p300 and CBP mutants was further confirmed by WB analysis of core histone proteins prepared from 293T cells transfected with wild-type or mutant CBP or p300 at high transfection efficiency (Figure 4e and f, also see Supplementary Figure S6B). As shown in Figure 4e, although expression of wild-type CBP led to increased levels of histone acetylation for H3, H2A/H2B and H4 in a CBP dose-dependent manner, expression of the I1432G mutant CBP did not result in increased histone acetylation. Importantly, while expression of the wild-type CBP led to increased histone crotonylation in a CBP dose-dependent manner, expression of the I1432G mutant CBP resulted in even stronger increases of histone crotonylation (Figure 4e). As CBP has also been shown to catalyze histone propionylation and butyrylation [20, 21], we also tested how the mutation affects histone propionylation and butyrylation. We found that expression of wild-type but not mutant CBP increased histone propionylation (Figure 4e). On the other hand, although expression of wild-type CBP did not significantly increase histone butyrylation, a strong increase of histone butyrylation was observed when the mutant CBP was expressed (Figure 4e). Thus, this novel CBP mutant has impaired both histone acetylation and propionylation activities but elevated both histone crotonylation and butyrylation activities. The same results were observed for p300 I1395G mutant (Figure 4f). Using pan-specific antibodies, we observed that p300 also catalyzed non-histone acetylation and crotonylation and the p300 I1395G was impaired in non-histone acetylation but was more active in non-histone crotonylation (Supplementary Figure S6B).

### *In vitro* characterization of p300 I1395G and CBP I1432G mutants

**Figure 5:**
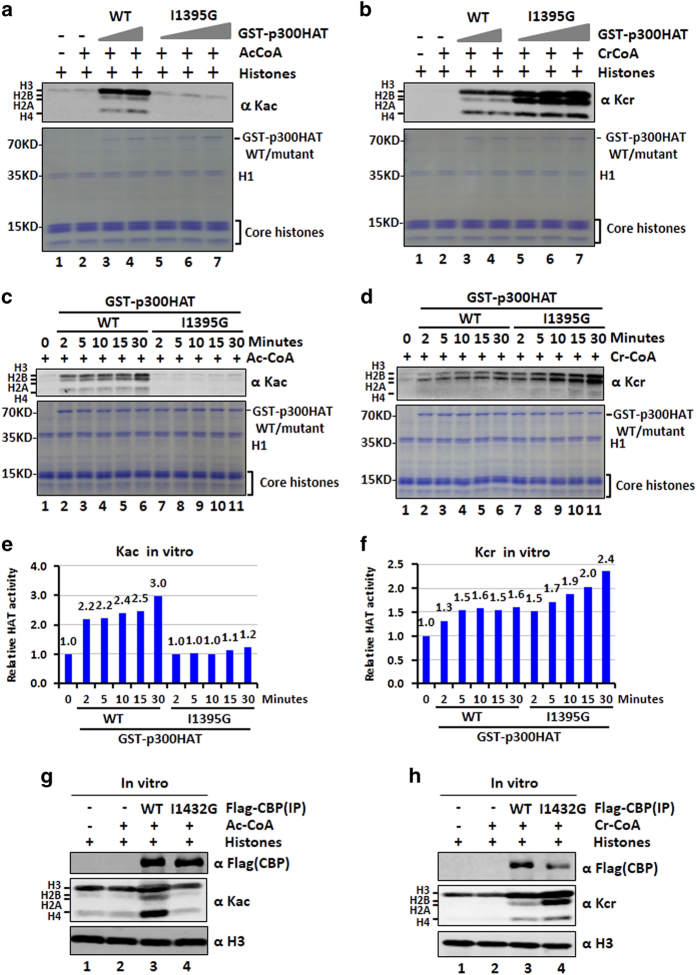
*In vitro* characterization of p300 I1395G and CBP I1432G mutant. (**a**) *In vitro* histone acetylation assay showing GST-p300-I1395G mutant is inactive in histone acetylation. Both wild-type and I1395G p300 histone acetyltransferase (HAT) domain were expressed and purified from bacteria and applied to HAT assay using core histones prepared from HeLa cells. The resulting histone acetylation was revealed by western blotting (WB) analysis using a pan-acetylated lysine antibody. Acetyl-coA, 100 μm. The components of *in vitro* acetylation reactions were also shown by Coomassie blue staining. (**b**) *In vitro* histone crotonylation assay showing that both wild-type GST-p300-HAT and GST-p300-I1395G mutant catalyzing histone crotonylation and that the mutant was more active. *In vitro* reactions were carried out using core histone substrates as above except crotonyl-coA (100 μm) was used. (**c**) The HAT activity of GST-p300-HAT and GST-p300-I1395G mutant was compared in a time course experiment. (**d**) The histone crotonyltransferase (HCT) activity of GST-p300-HAT and GST-p300-I1395G mutant was compared in a time course experiment. (**e**) The quantification of relative HAT activity of GST-p300-HAT and GST-p300-I1395G mutant according to results in **c**. (**f**) The quantification of relative HCT activity of GST-p300-HAT and GST-p300-I1395G mutant according to results in **d**. (**g**) *In vitro* histone acetylation assay using wild-type and I1432G mutant CBP immunoaffinity purified from 293T cells. (**h**) *In vitro* histone crotonylation assay using wild-type and I1432G mutant CBP immunoaffinity purified from 293T cells.

The availability of CBP/p300 mutants with preserved HCT but impaired histone acetylation and propionylation would significantly benefit the functional study of histone crotonylation. To ascertain the unique feature of these mutants, we expressed the HAT domain of wild-type p300 and the I1395G mutant as GST-fusion proteins and examined their HAT and HCT activities *in vitro* using core histones prepared from HeLa cells as substrates [36]. In agreement with the results of our cell-based assays, we found that while the recombinant wild-type p300 HAT strongly catalyzed histone H3 acetylation, the mutant exhibited poor, if any, HAT activity toward H3, H2A/H2B and H4 (Figure 5a). In contrast, while wild-type p300 exhibited a robust HCT activity toward H3, H2A/H2B and H4, the mutant was clearly more active than the wild-type for crotonylation of all four core histones (Figure 5b). Using similar *in vitro* assays, we found that the p300 HAT I1395G mutant was defective in catalyzing histone propionylation but highly active in catalyzing histone butyrylation (Supplementary Figure S5D and E). The impaired HAT and elevated HCT activity of the GST-p300-I1395G mutant was further demonstrated by *in vitro* enzymatic assay in a time course experiment (Figure 5c and d and their corresponding quantitative results in Figure 5e and f). Moreover, the impaired HAT and elevated HCT activities were also confirmed by *in vitro* histone acetylation assay (Figure 5g) and crotonylation assay (Figure 5h) using immunoaffinity-purified wild-type CBP and the I1432G mutant. Together, these *in vitro* acetylation and crotonylation assays demonstrate that the I1395G mutation in p300 and I1432G mutation in CBP result in a simultaneous loss of HAT and gain of HCT activity. As no competition between acetyl-CoA and crotonyl-CoA existed in this *in vitro* assay, we conclude that the elevated HCT activity is the intrinsic property of the p300 I1395G and CBP I1432G mutants.

### p300 I1395G and CBP I1432G mutants are active transcriptional coactivators

**Figure 6:**
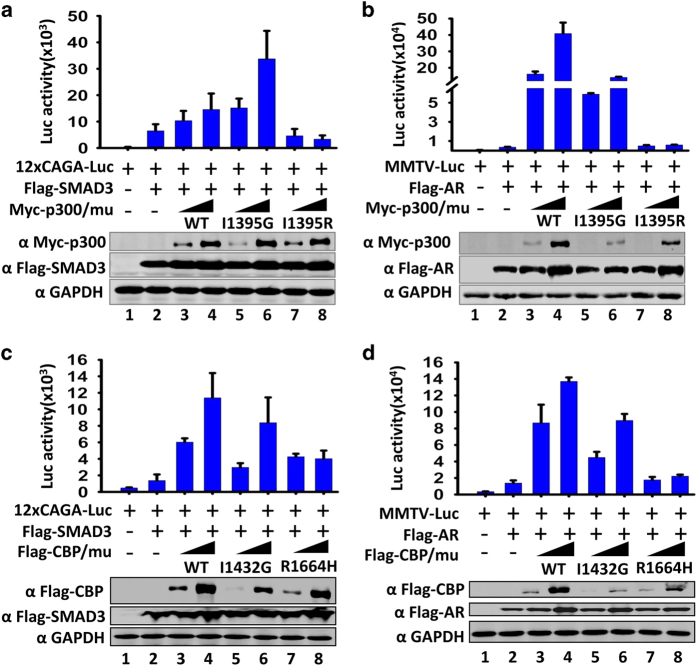
Both p300 I1395G and CBP I1432G mutants are functionally active transcriptional coactivators. (**a**) The p300 I1395G mutant but not the I1395R mutant enhanced transcriptional activation by SMAD3 in lucliferase reporter assay. Data are represented as relative luciferase activity (×10^3^)±s.d. of three technical duplicates. The expression of transfected Flag-SMAD3 and wild-type and p300 mutants was revealed by western blotting (WB) analysis. (**b**) The p300 I1395G mutant but not the I1395R mutant enhanced transcriptional activation by liganded AR in lucliferase reporter assay. Data are represented as relative luciferase activity (×10^4^)±s.d. of three technical duplicates. The cells were treated with 100 nm dihydrotestosterone (DHT) overnight before being collected for luciferase assay. (**c**) The CBP I1432G mutant but not the R1664H mutant enhanced transcriptional activation by SMAD3 in lucliferase reporter assay. Data are represented as relative luciferase activity (×10^3^)±s.d. of three technical duplicates. (**d**) The CBP I1432G mutant but not the R1664H mutant enhanced transcriptional activation by liganded AR in lucliferase reporter assay. The cells were treated with 100 nm DHT overnight. Data are represented as relative luciferase activity (×10^4^)±s.d. of three technical duplicates. All statistical analysis was performed using SigmaPlot 12.5.

The availability of p300 I1395G and CBP I1432G mutants allowed us to test the role of CBP/p300-catalyzed histone crotonylation/butyrylation in transcription in the absence of their catalyzed acetylation and propionylation. As general transcriptional coactivators, CBP and p300 have been shown to enhance transcriptional activation by various transcription factors including SMADs, androgen receptor, Sox2 and Oct4 [37,38,39,40,41,42].We thus first compared the ability for wild-type p300, the I1395G mutant and the I1395R mutant with loss of both HCT and HAT activities to enhance transcriptional activation by SMAD3 and androgen receptor using reporter assay. Representative results in Figure 6a and b show that wild-type p300 was able to substantially enhance transcriptional activation by SMAD3 and liganded AR, respectively, whereas the I1395R mutant was generally inactive or only marginally active. However, in all cases, the p300 I1395G mutant was nearly as active as or more active than the wild-type p300. Similarly, we found that the CBP I1432G mutant was also as active as the wild-type CBP in facilitating transcriptional activation by SMAD3 (Figure 6c) and liganded AR (Figure 6d), whereas the R1664H mutant with loss of both HAT and HCT activities failed to do so. Furthermore, we observed that the p300 I1395G mutant but not the I1395R mutant was as active as the wild-type p300 in facilitating transcriptional activation by SOX2 (Supplementary Figure S7A) and OCT4 (Supplementary Figure S7B). Similarly, the CBP I1432G but not R1664H mutant was able to support transcriptional activation by SOX2 (Supplementary Figure S7C) and OCT4 (Supplementary Figure S7D). These results collectively provide evidence that CBP and p300 are able to enhance transcriptional activation in cells in the absence of HAT activity, most likely through their remaining HCT activity.

### Transcriptional activation by the p300 I1395G mutant correlates with promoter histone crotonylation and recruitment of crotonylation reader DPF2

**Figure 7:**
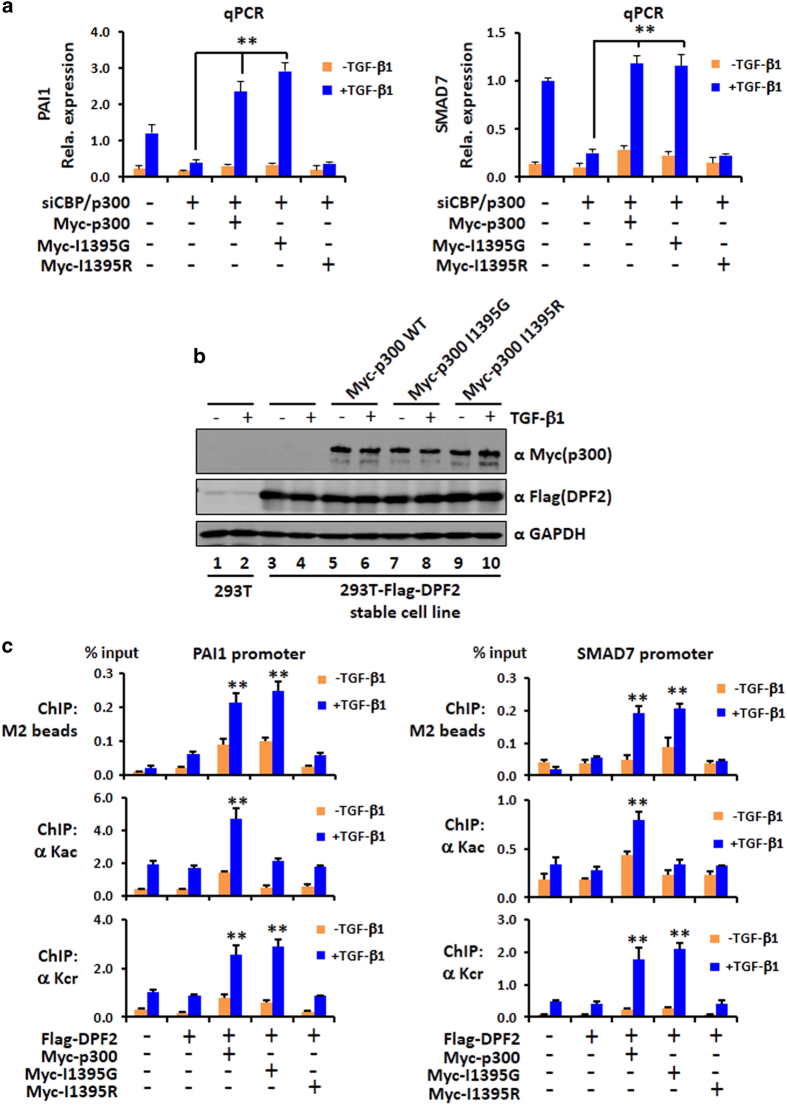
The p300 I1395G but not I1395R mutant supports TGF-β1-induced transcriptional activation, promoter histone crotonylation and recruitment of crotonylation reader DPF2. (**a**) Ectopic expression of the p300 I1395G but not the I1395R mutant was able to rescue TGF-β1-induced transcriptional activation of *PAI1* and *SMAD7* genes on knockdown of endogenous CBP and p300 by small interfering RNAs (siRNAs). 293T cells were treated with siRNAs targeting the 3′ noncoding regions of CBP and p300 mRNAs for 2 days before transfection with Myc-p300 or Myc-p300 mutants as indicated. One day after transfection, the cells were treated with TGF-β1 overnight before being collected for RT-PCR analysis. The levels of PAI1 and SMAD7 transcripts were determined by quantitative RT-PCR. Data are represented as relative level of transcript±s.d. of three technical duplicates. *P*-value ⩽0.01. (**b**) Western blotting (WB) analysis showing the expression of Myc-tagged wild-type or mutant p300 proteins in 293T cells stably expressing Flag-DPF2. (**c**) Chromatin immunoprecipitation (ChIP) assay showing that ectopic expression of I1395G mutant restored histone crotonylation but not acetylation and recruitment of crotonylation reader DPF2. The 293T cells stably expressing Flag-DPF2 were transfected without or with the wild-type, I1395G or I1395R mutants as indicated. Two days after transfection, the cells were treated with TGF-β1 overnight before being collected for ChIP analysis of the promoter regions of *PAI1* and *SMAD7* genes using pan-crotonylated lysine or pan-acetylated lysine antibodies. The recruitment of Flag-DPF2 was determined by ChIP assay using anti-Flag antibody. Data are represented as means of % input±s.d. of technical replicates. **P-value ⩽0.01.

To test the role of p300 I1395G mutant in transcriptional regulation in a more physiologically relevant context, we first established conditions that simultaneous knockdown of CBP and p300 by siRNAs significantly impaired TGF-β1-induced transcriptional activation of TGF-β1 target genes *PAI1* and *SMAD7* [43] (Figure 7a). We then tested whether ectopically expressed wild-type, I1395G and I1395R p300 mutants could rescue TGF-β1-induced transcriptional activation. We found that expression of wild-type p300 not only fully rescued but also further augmented TGF-β1-induced activation of PAI1 and SMAD7 (Figure 7a), presumably due to elevated levels of p300 proteins as a result of transfection. Significantly, we found that ectopic expression of the p300 I1395G but not the p300 I1395R mutant augmented TGF-β1-induced activation of PAI1 and SMAD7 (Figure 7a). Thus, the HAT activity-deficient p300 I1395G mutant is able to substitute endogenous CBP/p300 to enhance TGF-β1-induced gene activation.

To test whether transcriptional activation by I1395G mutant p300 is linked to histone crotonylation and recruitment of crotonylation reader proteins, we established a 293T cell line expressing Flag-tagged crotonylation reader DPF2 [28] (Figure 7b). We then transfected the stable Flag-DPF2 cells with Myc-tagged wild-type p300, I1395G or I1395R mutants, respectively, and treated the cells with or without TGF-β1 overnight. The expression of WT and p300 mutant proteins was confirmed by WB analysis (Figure 7b). Subsequent chromatin immunoprecipitation (ChIP) assay revealed that, while expression of wild-type p300 markedly enhanced the levels of histone acetylation and crotonylation on both the PAI1 promoter and the SMAD7 promoter (Figure 7c), expression of I1395G mutant only enhanced histone crotonylation but not acetylation on both promoters. Expression of I1395R mutant enhanced neither histone acetylation nor histone crotonylation. ChIP assay for Flag-DPF2 showed that expression of either wild-type p300 or the I1395G mutant substantially enhanced TGF-β1-induced recruitment of DPF2 to both promoters (Figure 7c). However, TGF-β1-induced recruitment of DPF2 was not observed when the I1395R mutant was expressed. Thus, the ability for I1395G mutant to enhance TGF-β1-induced gene expression correlates with its HCT activity and recruitment of crotonylation reader proteins DPF2. Together, these data confirm impaired HAT activity for the I1395G mutant and support the idea that CBP/p300 is able to enhance transcriptional activation through histone crotonylation.

## Discussion

Recent identification of multiple histone lysine acylation including propionylation, malonylation, butyrylation, succinylation and crotonylation has not only raised significant interests on cross talk between metabolism and epigenetic regulation but also their functions in transcription [15, 17, 19, 21]. A prerequisite toward the understanding of their biological functions is to define and characterize the enzymes catalyzing and removing these PTMs. In this study, we investigated within mammalian nuclear HATs the proteins that can catalyze histone crotonylation. Here we demonstrate that, in addition to CBP and p300, MOF also has HCT activity. We present evidence that MOF is an evolutionarily conserved HCT. We also present evidence that CBP and p300 are the major HCTs in mammalian cells. Importantly, by generating HAT-deficient and HCT-competent CBP and p300 mutants we were able to demonstrate that CBP and p300 can mediate transcriptional activation in cells in the absence of HAT activity. Thus, our study supports the emerging theme that histone acylation other than acetylation can have important roles in transcriptional regulation and possibly other processes as well [14, 44].

By IF staining and WB assays, we showed that ectopically expressed CBP and p300 are highly active for histone crotonylation (Figure 1a and b). Our finding that CBP and p300 are active HCTs is in full agreement with a recent work by David Allis and his colleagues [22]. In contrast, we detected no HCT activity for ectopically expressed GCN5, PCAF, HBO1 and TIP60 under the same condition (Supplementary Figure S2). The lack of HCT activity for these HATs is also consistent with the study by Sabari *et al.* [22]. However, we also detected a robust HCT activity for MOF (Figure 1a and b) and demonstrated that MOF can catalyze histone crotonylation at multiple sites on both H3 and H4 (Figure 2a). The discrepancy between ours and Sabari’s study is likely due to differences in methods for detection of HCT activity. *In vitro* HCT activity assay was used in their study, whereas cell-based IF staining was used in ours. It is noteworthy that we also failed to detect HCT activity *in vitro* for recombinant MOF (data not shown). The failure to detect a HCT activity for recombinant MOF is unlikely due to problem in protein preparation, because the same recombinant MOF displayed an HAT activity *in vitro* (data not shown). Similarly, we also failed to detect HCT activity for recombinant yeast Esa1, suggesting that both MOF and Esa1 most likely function as HCT in cells in the context of a protein complex [32, 33]. The identification of MOF and Esa1 as HCTs provides an explanation for evolutional conservation of histone crotonylation. Although CBP and p300 possess robust HCT activity, there is no CBP and p300 homolog in yeast. Our finding that MOF also possesses HCT activity promoted us to test and demonstrate that Esa1 is likely the enzyme responsible for bulk histone crotonylation in yeast (Figure 2c and d).

Despite our identification of MOF as an evolutionarily conserved HCT capable of catalyzing histone crotonylation at multiple sites, our study points to CBP and p300 as the enzymes responsible for bulk histone crotonylation in mammalian cells. Treatment with CBP/p300 selective inhibitor C646 resulted in drastic reduction of histone crotonylation and acetylation in various cell lines tested (Figure 3 and Supplementary Figure S4). Furthermore, knockdown of CBP and p300 by shRNAs also led to marked reduction of histone crotonylation as well as acetylation. The finding that CBP and p300 are the major HCTs in mammalian cells is consistent with CBP/p300 as the versatile acyltransferases that also catalyze histone propionylation and butyrylation [21, 23]. In this regard, it is tempting to suggest that CBP and p300 are evolved later in evolution to allow a more intimate coupling between metabolism and transcription. It is noteworthy that CBP and p300 also broadly catalyze crotonylation on non-histone proteins (Supplementary Figure S6), although the identities of these non-histone substrates remain to be determined.

Although histone propionylation, malonylation, butyrylation and crotonylation have been reported for a number of years, the exact function of these various types of PTMs remains to be defined. A major obstacle in studying these PTMs is the fact that they are also generated by the well-documented HAT enzymes such as CBP and p300. Among these PTMs, crotonylation attracts significant attention because it occurs broadly in histones [15]. Although the study by Sabari *et al*. showed that p300 could robustly enhance transcriptional activation *in vitro* in the presence of crotonyl-CoA, it is not clear whether CBP/p300 can facilitate transcriptional activation in cells through crotonylation, as the cellular crotonyl-CoA concentration was estimated to be two to three orders of magnitude lower than acetyl-CoA [22]. Thus, to define the function of histone crotonylation in cells, a CBP or p300 mutant with deficient HAT but preserved HCT activity is essential. Through mutagenizing the key isoleucine residue in the reported acetyl-CoA binding pocket, we have successfully obtained the CBP I1432G and p300 I1395G mutants that exhibit a deficient HAT but competent HCT activity. Further characterization indicates that these mutants are also deficient in histone propionylation and competent for histone butyrylation. The characteristics of these CBP/p300 isoleucine-to-glycine mutants are likely the result of favorable binding for acyl-CoA with larger sizes such as crotonyl-CoA and butyryl-CoA and unfavorable binding for acetyl-CoA and propinyl-CoA with a smaller size due to an increased size of the binding pocket. Using these mutants, we were able to demonstrate that CBP and p300 can enhance transcriptional activation by various transcription factors in an HAT activity-independent manner. Furthermore, we demonstrated these HAT-deficient p300 mutant was able to replace endogenous CBP/p300 to support TGF-β1-induced gene activation.

Although histone crotonylation and acetylation are catalyzed by the same enzymes (CBP, p300 and MOF), their distinct molecular structures suggest a non-redundant effect on chromatin structure and function. In support of this idea, distinct effector proteins have been identified to read histone crotonylation and acetylation. For example, the YEATS domains and double PHD finger proteins have been reported to selectively bind crotonylated histone tails and a role for AF9 YEATS domain in positive regulation of transcription has been demonstrated [26, 29]. Consistent with these findings, we demonstrate that transcriptional activation by the HAT-deficient and HCT-competent p300 mutant correlates with elevated promoter histone crotonyation and recruitment of crotonylation reader protein DPF2 [29], a subunit of BAF chromatin remodeling complex [12, 13]. Thus, it is tempting to suggest that histone crotonylation catalyzed by CBP, p300 and MOF is likely to facilitate transcriptional activation through recruitment of crotonylation reader proteins such as DPF2, which in turn facilitates transcription by chromatin remodeling. However, as CBP I1432G and p300 I1395G mutants also catalyze histone butyrylation, it is formally possible that histone butyrylation (potentially additional acylation as well) also contributes to the transcriptional activation enhanced by HAT-less CBP/p300. Nevertheless, the novel CBP/p300 mutants identified in our study will be valuable for elucidating the molecular mechanism and function of histone crotonylation and/or butyrylation and their reader proteins.

## Materials and methods

### Cell lines, antibodies, reagents and plasmids

HeLa S3 and 293T cells were cultured in Dulbecco’s Modified Eagle’s medium (GIBCO, Thermo Fisher, Waltham, MA, USA) with 10% fetal bovine serum (GIBCO) at 37 °C in a 5% CO_2_ in air atmosphere. The following antibodies were used in this study: pan-Kac (PTM-Biolabs 101, Shanghai, China), pan-Kcr (PTM-Biolabs 501), pan-Kpr (PTM-Biolabs 201), H3K9cr (PTM-Biolabs 516), H3K18cr (PTM-Biolabs 517), p300 (Carlsbad, CA, USA) (Active Motif 61401).

H3 (Epitomics M1309-1, Hangzhou, China), Flag (Sigma 7425/1804, St Louis, MO, USA), and Myc (Abmart 20002 mouse, Shanghai, China). CBP rabbit polyclone antibody was homemade. CBP/p300 inhibitor C646 was from Sigma (SML0002) and was used in final concentration 20 or 40 μm. TGF-β1 was added to a final concentration of 2 ng ml^−1^ overnight after cultured HEK293T cells 16 h in no fetal bovine serum Dulbecco’s Modified Eagle’s medium (GIBCO). The pCMV-Flag-CBP, pCMV- p300-Myc, pCMV-Flag-TIP60, pCMV-Flag-PCAF, pcDNA3.1-Flag-HBO1, pcDNA3.1-Flag-GCN5 and pcDNA3.1-MOF were either as described or constructed by our lab [45]. All mutants were generated by PCR-based point mutagenesis strategy and verified by DNA sequencing. Lipofectamine 2000 (Thermo Fisher) was used for the transfection of nucleic acids (DNA and RNA) into eukaryotic cells according to manufacturer’s instructions.

### Yeast strains culture and histone preparation

All budding yeast strains used in this study were derived from yeast strain BY4742, and yeast cells were cultured in YPD (10 g l^−1^ yeast extract, 20 g l^−1^ peptone, 2% dextrose) with or without sodium crotonate at 30 °C. Acetyltransferase knock-in or knockout strains were established as described [46]. Histone preparation from budding yeast was also as described [46].

### Western blot analysis and Immumofluorescent staining

For western blot analysis of histones, histones were purified from mammalian cells using a standard acid extraction protocol [47]. WB analyses for various proteins including histones was carried out using standard protocol. In brief, the proteins samples were separated on SDS-PAGE gels (8% for other proteins and 15% for histones) and then transferred to polyvinylidene fluoride membranes. The membranes were blocked in 5% milk diluted in a mixture of Tris-buffered saline and Polysorbate 20 (TBST; containing 0.1% Tween) for at least 1 h at room temperature or overnight at 4 °C with rocking and then washed with TBST. The membranes were incubated in primary antibody either overnight at 4 °C or at room temperature for 1–2 h with rocking. The blots were washed with TBST and then incubated for 1 h with rocking in Li-Cor secondary antibodies used at a concentration of 1:10 000 in TBST. Western blots were developed using a Li-Cor Odyssey.

Immumofluorescent staining was performed essentially as described [48]. In brief, HeLa cells were seeded on coverslips before experiment. The cells were pre-washed with phosphate-buffered saline (PBS) twice and fixed in 4% paraformaldehyde for 15 min at room temperature. After brief rinse with PBS three times, the cells were permeablized with 1% Triton X-100 in PBS for 15 min on ice followed by PBS rinse again. The permeabilized cells were then blocked with 5% BSA in PBST (PBS with 0.2% Triton X-100) for 30 min at room temperature. After brief rinse with PBST for three times, cells were incubated for 2 h at 37 °C or overnight at 4 °C with relevant antibodies in appropriate concentration depending of antibodies’ titer. After rinsing with PBST three times, the cells were incubated with Alexa Fluor-conjugated goat anti-rabbit or anti-mouse IgG for 1 h at 37 °C. Finally, the cells were counterstained with DAPI and mounted onto glass slides with Vectashield mounting medium (Vector Laboratories). Microscopic images were captured by confocal microscope (Leica TCS SP5 II, Buffalo Grove, IL, USA).

### Preparation of core histones for *in vitro* HAT/HCT assays from HeLa cells

To prepare core histones with low basal acetylation and crotonylation, HeLa cells were cultured with PBS for 2 h before being collected. HeLa S3 (~2×10^7^) cells were collected by scrape and then washed in cold PBS. The pellet was resuspended in hypotonic lysis buffer (10 mm Tris-HCl pH 7.9, 1 mm KCl, 1.5 mm MgCl_2_ and 1 mm DTT) and incubated for 30 min on rotator at 4 °C to promote hypotonic swelling of cells. The intact nuclei were collected by centrifugation at a speed of 13 000 r.p.m. for 15 min at 4 °C. The pellets were resuspended in 0.4 n H_2_SO_4_ and then incubated on rotator at 4 °C for 2 h. The samples were then clarified by centrifugation at 13 000 r.p.m. for 1 h. The supernatants were then subjected to TCA precipitation of histones (final concentration of TCA 33%) and incubated on ice for 30 min. After centrifugation at 13 000 r.p.m. for 15 min at 4 °C, the precipitates were collected and washed with ice-cold acetone twice. The pellets were dissolved in an appropriate volume of ddH_2_O after air-dry histone pellet for 20 min at room temperature. The resulting core histones can be used as substrate for *in vitro* HAT/HCT assays.

### Preparation of recombinant GST-p300 HAT proteins

GST-tagged wild-type or mutant human p300 HAT domains (1 285–1 664) were constructed based on the pGEX-4T-1 vector. GST-p300-HAT-WT/I1395G were expressed in *Escherichia coli* strain Rosetta (DE3) (Cwbio) and induced with 0.2 mm IPTG overnight at 16 °C. After cell lysis by using an AH-1500 (ATS) high-pressure homogenizer and centrifugation, the supernatants were applied to Glutatione Sepharose (GE Healthcare, Little Chalfont, UK)-based affinity purification according to the manufacturer’s instructions.

### *In vitro* HAT/HCT assays

For *in vitro* HAT and HCT assays, wild-type or mutant GST-p300 HAT proteins were expressed and purified from *E. coli* as described above. HAT and HCT assays were carried out at 37 °C for 2 min to 1 h in Buffer A (25 mm Tris pH 8.0, 150 mm NaCl and 1 mm DTT) with 100 μm crotonyl-CoA or acetyl-CoA (Sigma 28007 and A2181) and 2 μg core histones prepared from HeLa cells. Histone acetylation and crotonylation were then detected by WB analysis using various antibodies as indicated.

### Chromatin immunoprecipitation assay

ChIP assays were performed essentially as described [49]. In brief, the cells (1×10^7^) were cross-linked directly on plate with 1% formaldehyde (in PBS) for 10 min with gentle shaking. Glycine was added to a final concentration of 1.25 m to quench the cross-linking and allowed to incubate for 5 min. The cells were scraped off the plates, pelleted and washed in PBS plus 1× Complete protease inhibitors (Sigma). The pellets were resuspended to 1 ml Solution A (10 mm Tris-HCl, pH 7.9; 0.25% Triton X-100; 10 mm EDTA; protease inhibitors) and incubated with rotation at 4 °C for 10 min. The samples were centrifuged and the pellets were resuspended in 600 μl Solution C (1 mm EDTA; 150 mm NaCl; 50 mm Tris-HCl pH 7.5; 0.1% SDS; 1% Triton X-100; 0.1% sodium deoxycholate; 2 mm PMSF; protease inhibitors). The samples were then sonicated using Bioruptor Plus (Diagenode, Denville, NJ, USA) for 1 h with the following settings: high power, work 5 s, rest 10 s. The samples were clarified by centrifugation at 16 000 *g* for 15 min. The resulting supernatants (soluble chromatin extract) were used for ChIP analysis as follow: 100 μl of soluble chromatin, 2 μg of specific antibody and 8 μl of 50% slurry of protein A or G (depending on the source of antibody) equilibrated with ChIP I buffer (0.1% sodium deoxycholate; 1% Triton X-100; 2 mm EDTA; 50 mm Tris-HCl, pH 7.5; 150 mm NaCl; 1 mm DTT) containing salmon sperm DNA (200 μg ml^−1^) and BSA (1 mg ml^−1^), and ChIP I buffer to a final volume of 400 μl. The ChIP reactions were incubated overnight with rotation at 4 °C. For ChIP against FLAG tag, FLAG-M2-beads (SIGMA F1804) were added to diluted extract and incubated overnight with rotation at 4 °C. The samples were centrifuged at 3 000 r.p.m. for 1 min at 4 °C to pellet beads. The beads were washed sequentially for 10 min at 4 °C with 400 μl of each of the buffers listed below: ChIP I buffer, ChIP II buffer (0.1% sodium deoxycholate; 1% Triton X-100; 2 mm EDTA; 50 mm Tris-HCl, pH 7.5; 500 mm NaCl; 1 mm DTT), ChIP III buffer (0.25 m LiCl; 0.5% NP-40; 0.5% sodium deoxycholate; 1 mm EDTA; 10 mm Tris-HCl, pH 8.0; 1 mm DTT) and TE buffer (10 mm Tris-HCl, pH 8.0; 1 mm EDTA; 1 mm DTT). The washed beads were resuspended in 300 μl of fresh elution buffer (0.5% SDS; 0.1 m NaHCO_3_) plus 0.5 μl of Proteinase K (10 mg ml^−1^) and 0.5 μl RNaseA (10 mg ml^−1^). The samples were vortexed briefly and incubated at 65 °C for 6 h or overnight to reverse cross-link. The samples were extracted with 100 μl of phenol/chloroform and DNAs were precipitated with ethanol and dissolved in appropriate volumes of H_2_O for quantitative PCR.

### Quantitative RT-PCR, shRNAs, siRNAs and luciferase reporter assay

Total RNA was extracted using the RNAiso Plus (Takara, Dalian, China, D9108A). All cDNAs were prepared using the TransScript One-Step gDNA Removal and cDNA Synthesis SuperMix (TransGen Biotech, Beijing, China, AT311) according to manufacturer’s instructions. All quantitative PCRs were performed using TransStart Tip Green quantitative PCR SuperMix (TransGen Biotech, AQ141) and CFX96 Touch Real-Time PCR Detection System (Bio-Rad, Hercules, CA, USA). The primers for RT-PCR are listed in Supplementary Table S1. Also provided in Supplementary Table S1 are sequences for siRNAs and/or shRNAs. The CBP/p300 siRNAs were targeted to the 3′-untranslated regions, which allowed overexpression of exogenous p300 wild-type or mutants plasmids upon knockdown of endogenous CBP/p300. All RT-PCR statistical analyses were performed by Comparative Delta-delta Ct method using Excel. Luciferase reporter assay was performed using a dual luciferase reporter assay kit from Promega. All luciferase reporter assay statistical analysis was performed using SigmaPlot 12.5.
