# Supplementary material for: MOF as an evolutionarily conserved histone crotonyltransferase and transcriptional activation by histone acetyltransferase-deficient and crotonyltransferase-competent CBP/p300
Source: Cell Discov. 2017 May 23;3:17016–. doi: 10.1038/celldisc.2017.16 (PMC5441097; doi:10.1038/celldisc.2017.16)
Supplement: Supplementary information [file celldisc201716-s1.pdf]

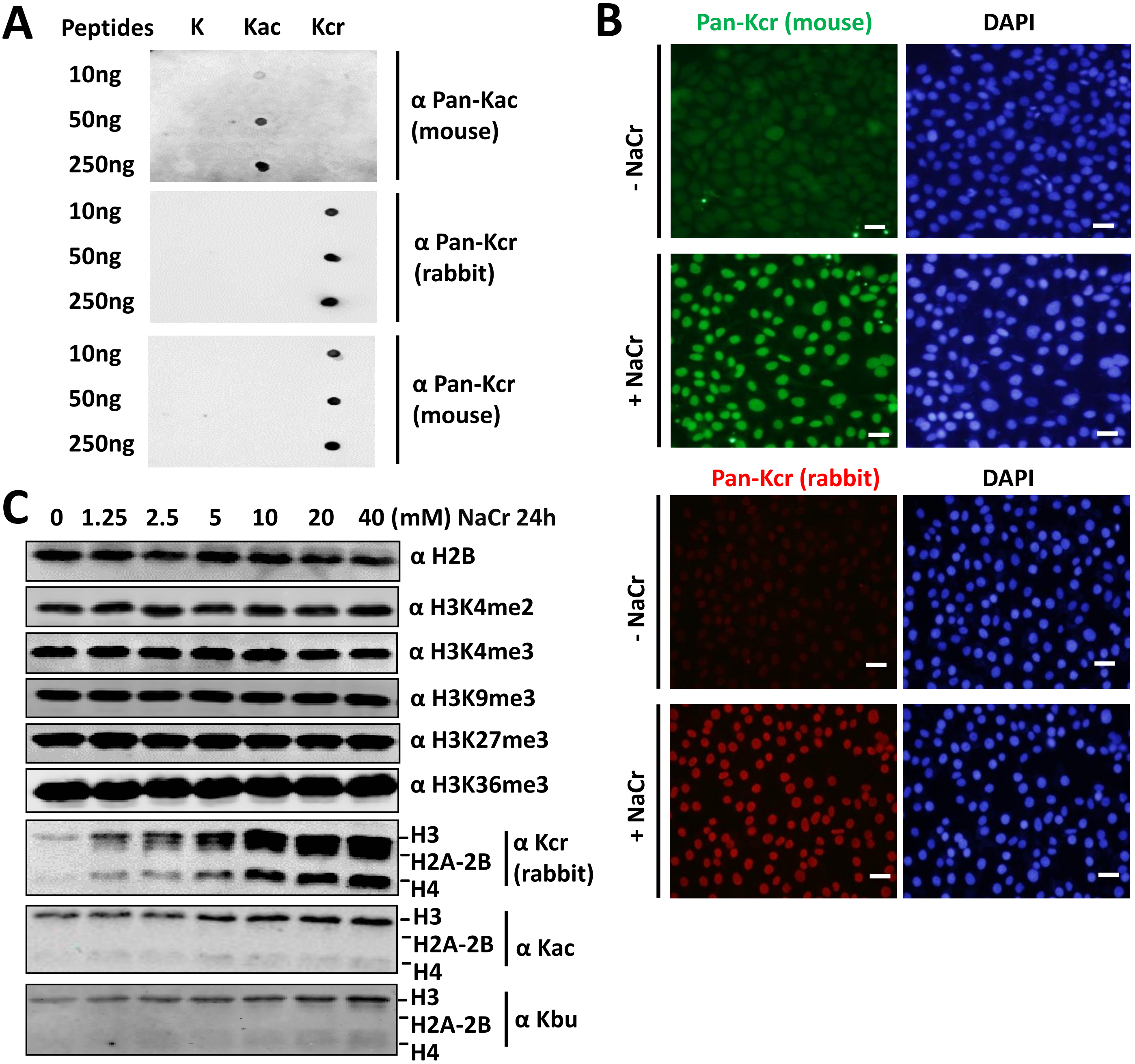

Supplementary Figure S1. Characterization of pan-Kcr antibodies. **(A)** Dot blot analysis showing that the pan-Kcr monoclonal and polyclonal antibodies were specific to crotonylation and showed no cross-reaction with acetylation. The synthetic unmodified H3, the H3 with K9, K14 crotonated or acetylated peptides (aa 1-27) were used at concentrations as indicated. Dilution of antibodies at 1:1000. **(B)** HeLa cells treated with or without sodium crotonate (NaCr) at a final concentration of 10 mM for 24 h were analyzed by immunostaining using the monoclonal pan-Kcr antibody at a dilution of 1:500 and polyclonal pan-Kcr antibody (1:1000) as indicated. Scale bar: 60 μm. **(C)** HeLa cells were treated with various concentrations of NaCr for 24 h and core histones were prepared and subjected to WB analysis using antibodies as indicated. WB for histone H2B served as loading control. Note NaCr treatment only results in substantial increases of histone crotonylation (Cell Research 2017; e-pub ahead of print 12 May 2017; doi:10.1038/cr.2017.68).

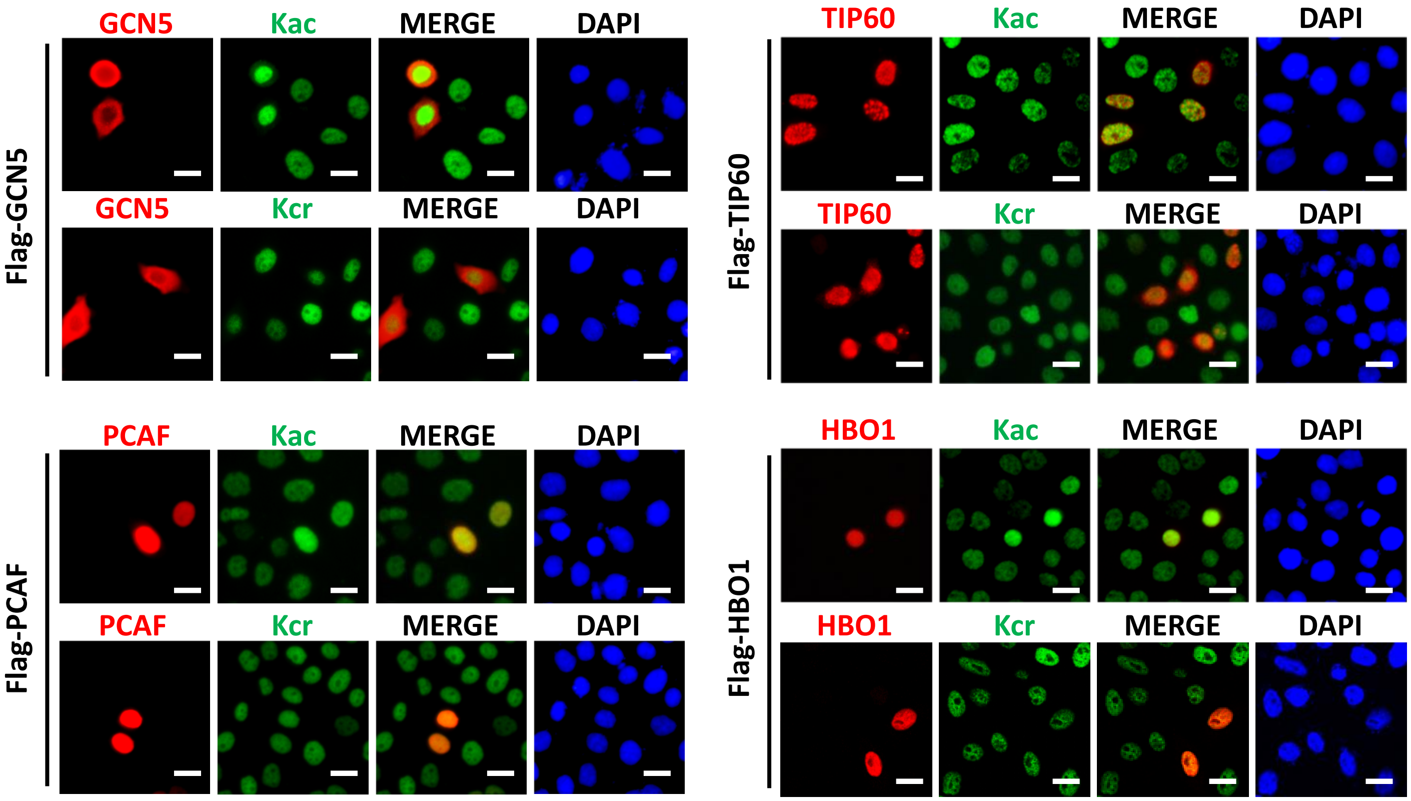

Supplementary Figure S2. Lack of HCT activity for GCN5, PCAF, TIP60 and HBO1. The above nuclear HATs were ectopically expressed in HeLa cells and the HAT and HCT activities were detected by immunostaining using pan-Kac or pan-Kcr antibodies, respectively. Scale bar: 20 μm. Note that each HAT was active for HAT activity.

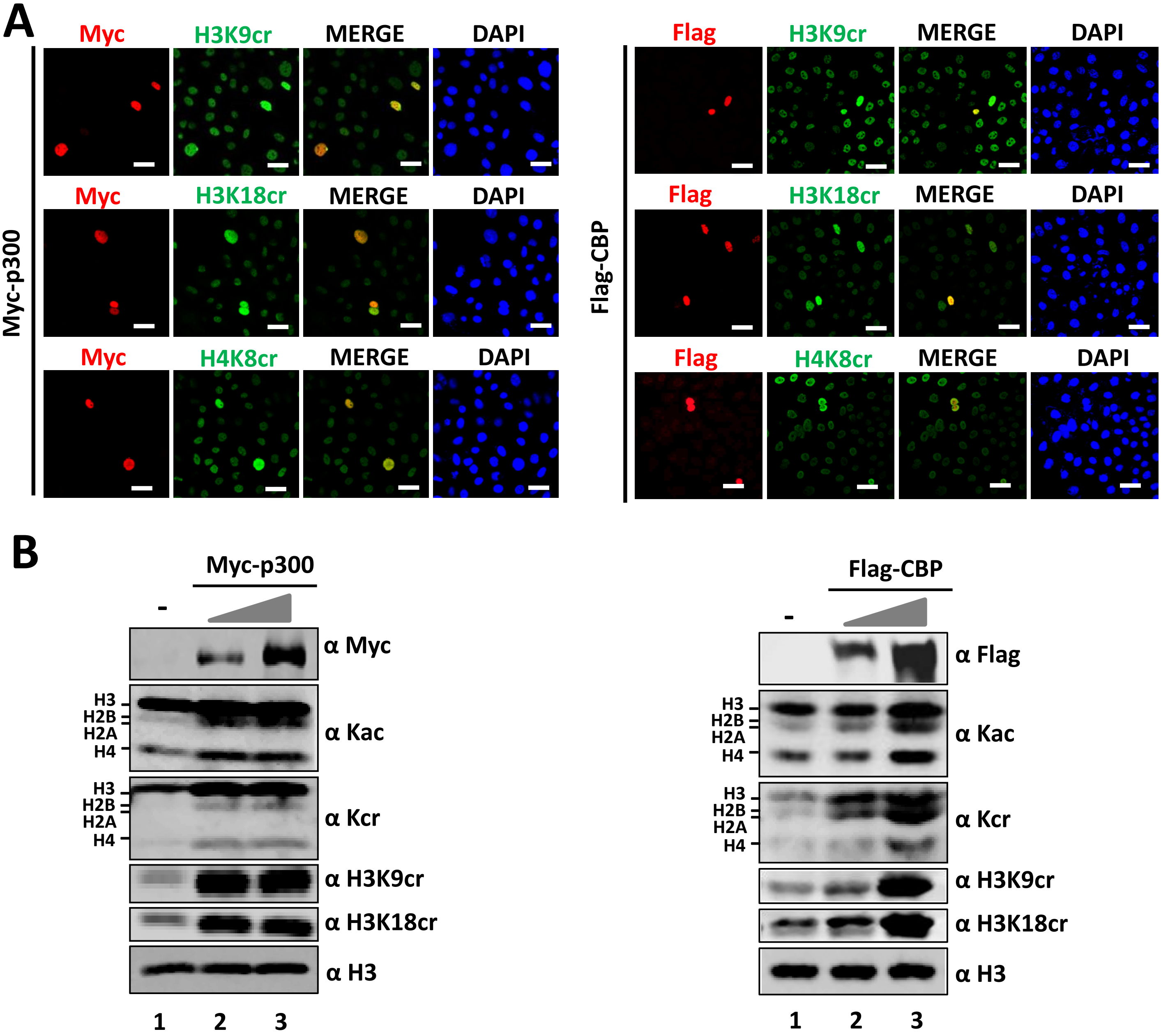

Supplementary Figure S3. CBP and p300 catalyze histone crotonylation at multiple sites. **(A)** Myc-p300 or Flag-CBP was ectopically expressed in HeLa cells and the ability to catalyze histone crotonylation was examined by immunostaining using site-specific histone crotonylation antibodies as indicated. Scale bar: 40 μm. **(B)** HeLa cells were transfected with Myc-p300 or Flag-CBP at high transfection efficiency condition (more than 90% cells transfected) and two days after transfection the whole cell extracts and core histones were prepared for WB analysis of Myc-p300 or Flag-CBP expression and histone crotonylation and acetylation, respectively. WB for histone H3 served as loading control.

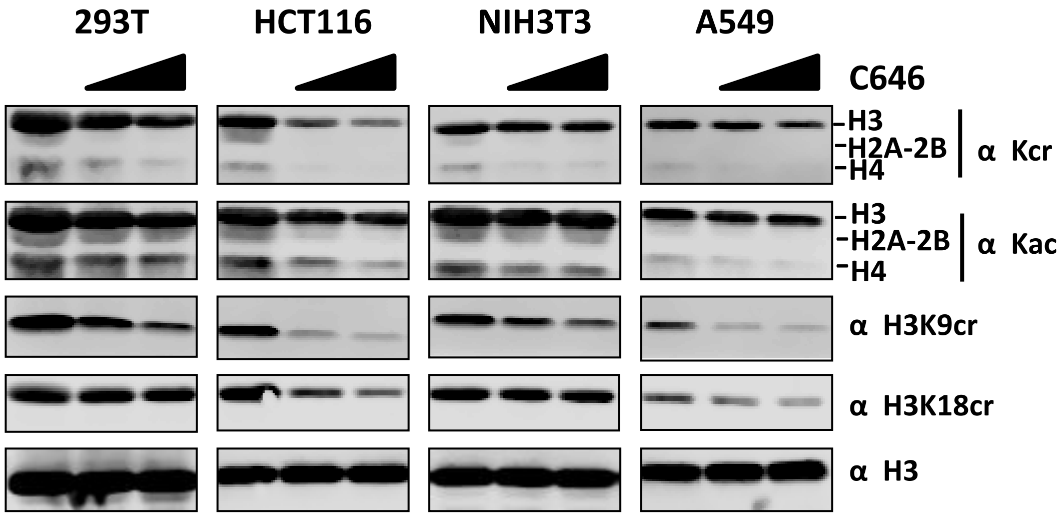

Supplementary Figure S4. The treatment with CBP/p300-selective inhibitor C646 results in substantial reduction of both histone acetylation and crotonylation in mammalian cells. The 293T, HCT116, NIH3T3 and A549 cells were treated with 0, 20 μM and 40 μM of C646 for 12h before harvested for preparation of core histones. The subsequent WB analysis was performed using pan-Kcr or site-specific H3Kcr antibodies as indicated. WB for histone H3 served as loading control.

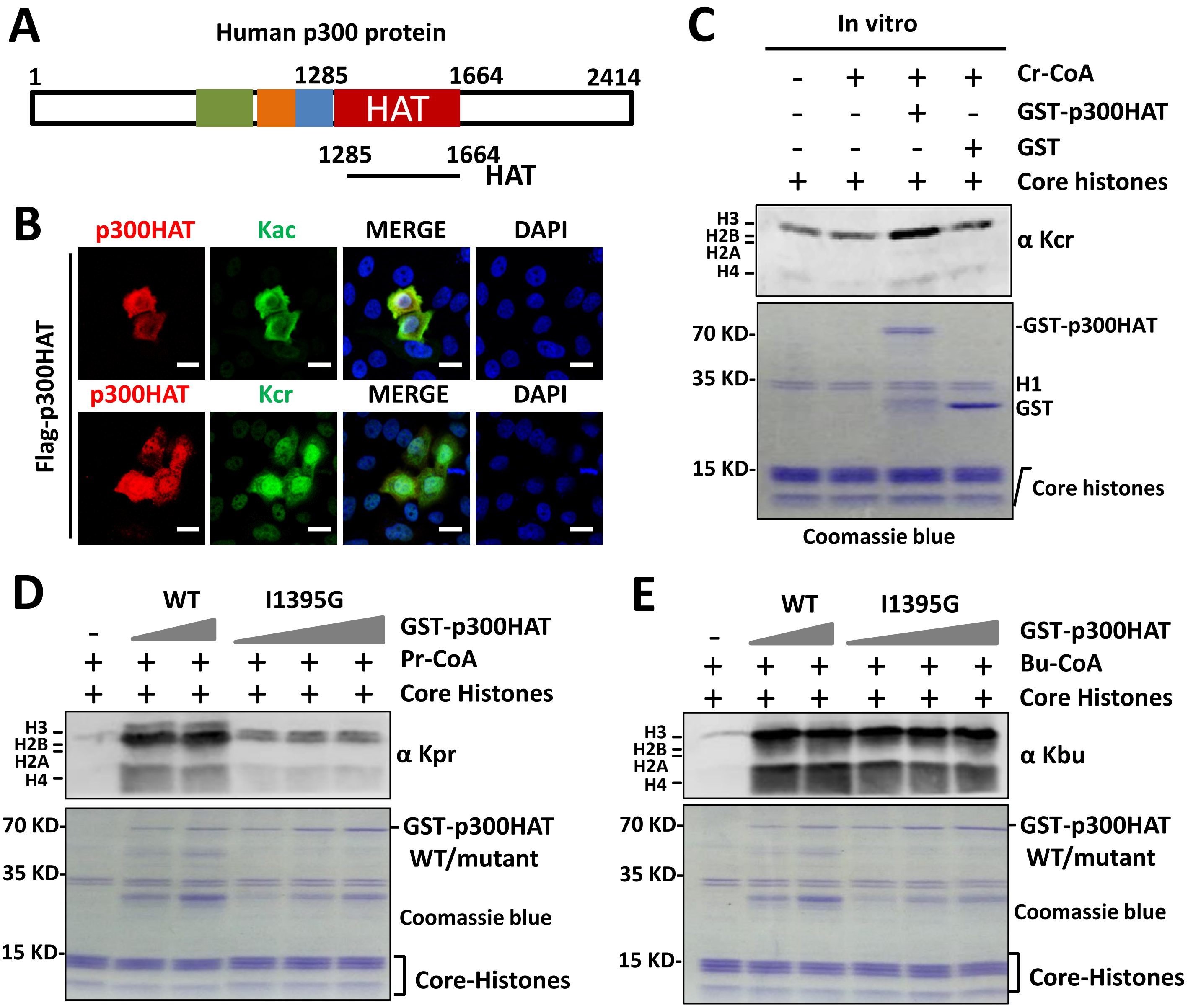

Supplementary Figure S5. The p300 I1395G mutant is defective in catalyzing histone propionylation but active for histone butyrylation *in vitro*. (A) Diagram illustrating the domain organization of human p300. HAT, histone acetyltransferase domain. **(B)** The p300 HAT domain is sufficient for catalyzing histone crotonylation in mammalian cells. HeLa cells were transfected with Flag-tagged p300 HAT domain and processed for immunostaining using pan-Kac and pan-Kcr monoclonal antibody, respectively. Scale bar: 20 μm. **(C)** *In vitro* histone crotonylation assay was performed with recombinant GST-p300 HAT and core histones prepared from HeLa cells. The crotonylation was detected by WB. Concentration of crotonyl-coA (Cr-CoA), 100 μM. Also shown was coomassie blue staining gel of the corresponding *in vitro* reactions. **(D)** *In vitro* histone propionylation assay was performed with recombinant GST-p300 HAT or GST-p300HAT-I1395G mutant and core histones prepared from HeLa cells. Concentration of propionyl-coA (Pr-CoA), 100 μM. **(E)** *In vitro* histone butyrylation assay was performed with recombinant GST-p300 HAT or GST-p300HAT-I1395G mutant and core histones prepared from HeLa cells. Concentration of butyryl-coA (Bu-CoA), 100 μM.

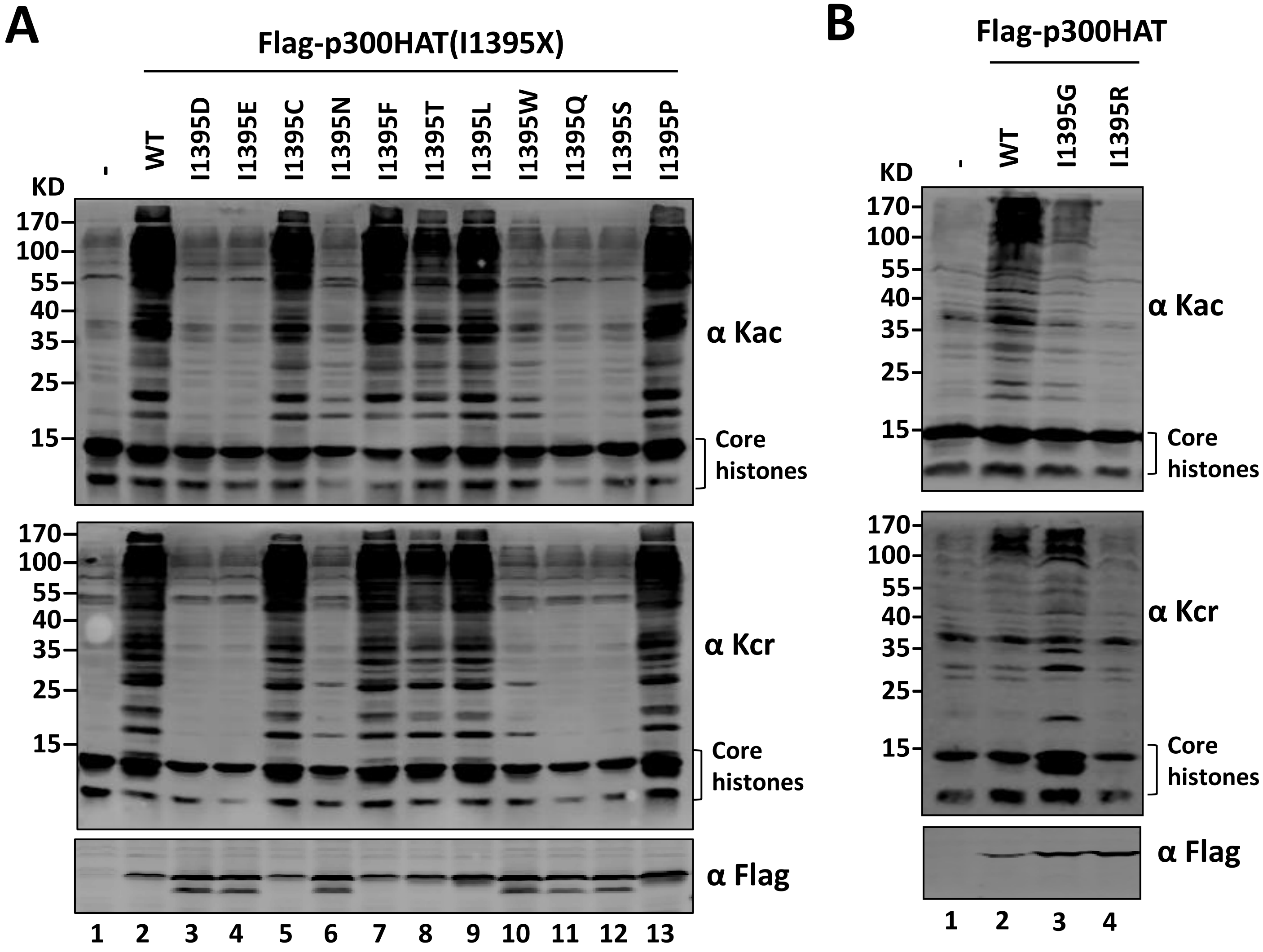

Supplementary Figure S6. Histone and non-histone crotonylation by p300 and various I1395 mutants. **(A)** The 293T cells were transfected with or without the wild-type and various p300 I1395 mutants as indicated. The whole cellular extracts were prepared 48h after transfection and subjected to WB analysis using pan-Kcr or pan-Kac antibodies as indicated. The expression of transfected wild-type or mutant p300 was verified by Western blot analysis using anti-Flag antibody. Note that the ability for p300 and its mutants to catalyze non-histone crotonylation parallels with the ability to catalyze histone crotonylation. **(B)** The 293T cells were transfected with or without the wild-type, I1395G and I1395R mutants as indicated. The WB analysis for histone- and non-histone-crotonylation was performed as in (A). Note the enhanced levels of crotonylation for the I1395G mutant.

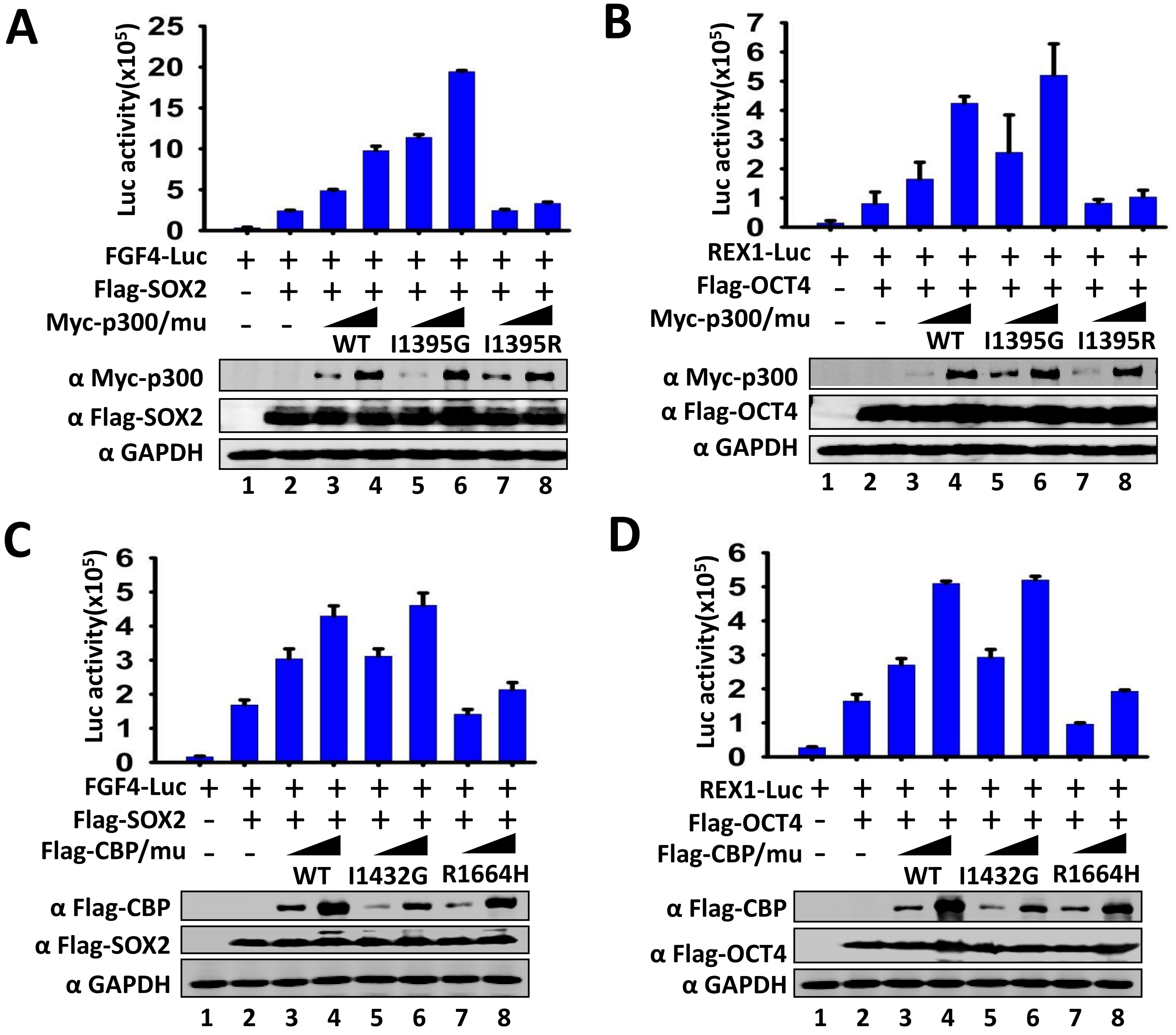

Supplementary Figure S7. The p300 I1395G and CBP I1432G mutants are active coactivators for Sox2 and Oct4. **(A-B)** The p300 I1395G mutant was able to enhance transcriptional activation by Sox2 **(A)** and Oct4 **(B)**. Amounts of the WT or mutant p300: 200 ng and 400 ng. Note the I1395R mutant defective in both HAT and HCT activities were inactive in facilitating Sox2 and Oct4 transcriptional activation. **(C-D**) The experiments were essentially as in **(A)** and **(B)** except the wild-type and CBP I1432G mutant were used instead of p300. Data are represented as relative luciferase activity ($x10^5$)±SD of three technical duplicates. All statistical analysis was performed using SigmaPlot 12.5.

Table S1

RT-PCR		forward	reverse
	PAI1	ACCGCAACGTGGTTTTCTCA	TTGAATCCCATAGCTGTTGAAT
	SMAD7	CTCCAGATACCCGATGGATTTTC	GCATCTGGACAGTCTGCAGTTG
	Actin	ATGGGTCAGAAGGATTCCTATGT	AGCCACACGCAGCTCATT

CHIP-qPCR			
	PAI1.4	CACAGAGAGAGTCTGGACA	CAACAGAGGACTCTTGGTCT
	SMAD7.3	GAGAGCCTTCTTATTTTGCC	GAATTCCGCGCACACGAAGA

siRNA		sense (5'-3')
	CBP	GAAGGGUGGAUUGAUGUU
	p300	AUGAACCUGAGGGAUGAU
	MOF	GAAAGAGAUCUACCGCAA

shRNA		sense (5'-3')
	CBP-1	CGCGAATGACAACACAGATTT
	CBP-2	CGGAGTCATCTAGTCCATAAA
	p300-1	GCCTTCACAATTCCGAGACAT
	p300-2	CCCGGTGAACTCTCCTATAAT
